# Global analysis of community-associated methicillin-resistant *Staphylococcus aureus* exoproteins reveals molecules produced *in vitro* and during infection

**DOI:** 10.1111/j.1462-5822.2006.00858.x

**Published:** 2007-05-01

**Authors:** Christopher Burlak, Carl H Hammer, Mary-Ann Robinson, Adeline R Whitney, Martin J McGavin, Barry N Kreiswirth, Frank R DeLeo

**Affiliations:** 1 Laboratory of Human Bacterial Pathogenesis, Rocky Mountain Laboratories, National Institute of Allergy and Infectious Diseases, National Institutes of Health Hamilton, MT 59840, USA.; 2 Reseach Technologies Branch, Mass Spectrometry Laboratory, National Institute of Allergy and Infectious Diseases, National Institutes of Health Rockville, MD 20852, USA.; 3 University of Toronto, Department of Laboratory Medicine and Pathobiology, and Sunnybrook Health Sciences Centre Toronto, ON, Canada, M4N 3M5.; 4 Public Health Research Institute Tuberculosis Center, International Center for Public Health Newark, NJ 07103, USA.

## Abstract

Community-associated methicillin-resistant *Staphylococcus aureus* (CA-MRSA) is a threat to human health worldwide. Although progress has been made, mechanisms of CA-MRSA pathogenesis are poorly understood and a comprehensive analysis of CA-MRSA exoproteins has not been conducted. To address that deficiency, we used proteomics to identify exoproteins made by MW2 (USA400) and LAC (USA300) during growth *in vitro*. Two hundred and fifty unique exoproteins were identified by 2-dimensional gel electrophoresis coupled with automated direct infusion-tandem mass spectrometry (ADI-MS/MS) analysis. Eleven known virulence-related exoproteins differed in abundance between the strains, including alpha-haemolysin (Hla), collagen adhesin (Cna), staphylokinase (Sak), coagulase (Coa), lipase (Lip), enterotoxin C3 (Sec3), enterotoxin Q (Seq), V8 protease (SspA) and cysteine protease (SspB). Mice infected with MW2 or LAC produced antibodies specific for known or putative virulence factors, such as autolysin (Atl), Cna, Ear, ferritin (Ftn), Lip, 1-phosphatidylinositol phosphodiesterase (Plc), Sak, Sec3 and SspB, indicating the exoproteins are made during infection *in vivo*. We used confocal microscopy to demonstrate aureolysin (Aur), Hla, SspA and SspB are produced following phagocytosis by human neutrophils, thereby linking exoprotein production *in vitro* with that during host–pathogen interaction. We conclude that the exoproteins identified herein likely account in part for the success of CA-MRSA as a human pathogen.

## Introduction

*Staphylococcus aureus* causes a wide range of human diseases, including impetigo, cellulitis, food poisoning, toxic–shock syndrome, necrotizing pneumonia, endocarditis and sepsis (Lowy, 1998; Diekema *et al*., 2001). Decades of selective pressure with β-lactam antibiotics and close proximity of susceptible hosts have resulted in a high prevalence of methicillin-resistant *S. aureus* (MRSA) in hospitals worldwide (Chambers, 2001; Diekema *et al*., 2001). Although these factors logically explain the high incidence of hospital-associated MRSA infections, the molecular basis for the increased incidence and severity of community-acquired (or associated) MRSA (CA-MRSA) infections among healthy individuals remains incompletely defined (Chambers, 2001; 2005; McDougal *et al*., 2003; Fridkin *et al*., 2005; Miller *et al*., 2005; Zetola *et al*., 2005). Recent studies indicate strains that are the leading causes of CA-MRSA disease in the United States, represented by pulsed-field gel electrophoresis (PFGE) types USA300-0114 (McDougal *et al*., 2003; Fridkin *et al*., 2005; Kazakova *et al*., 2005; Diep *et al*., 2006) and USA400 (Centers for Disease Control and Prevention, 1999; Baba *et al*., 2002; McDougal *et al*., 2003; Adem *et al*., 2005), have enhanced virulence compared with leading causes of hospital infections (e.g. USA200) (Voyich *et al*., 2005). In addition to their distinct PFGE profiles (McDougal *et al*., 2003), these two CA-MRSA strains can be distinguished from one another and from other *S. aureus* strains on the basis of multilocus sequence typing (MLST or ST) and sequencing of the variable number tandem repeats in the staphylococcal protein A gene (*spa*); USA300-0114 is *spa*-type 1 (Shopsin *et al*., 1999) and multilocus sequence type ST8, while USA400 is ST1 (Enright *et al*., 2000). Both strains also have the type IV staphylococcal chromosomal cassette *mec* element, which is common among CA-MRSA but not typically found in hospital adapted nosocomial MRSA (Baba *et al*., 2002; Voyich *et al*., 2005; Diep *et al*., 2006). In addition, each strain has one or more common names, such as Los Angeles County clone (LAC) or FPR3757 (sequenced strain) used for USA300-0114 (Voyich *et al*., 2005; Diep *et al*., 2006), and MW2 for the prototype USA400 strain (Centers for Disease Control and Prevention, 1999; Baba *et al*., 2002). Enhanced virulence of USA300-0114 (referred to herein as either LAC or USA300) and USA400 (referred to herein as MW2) is linked in part to their ability to circumvent killing by neutrophils and cause host cell lysis (Voyich *et al*., 2005). It is likely that exoproteins (cell surface-associated and freely secreted proteins) produced by these strains are an important component of this enhanced virulence (Foster, 2005; Voyich *et al*., 2005; Diep *et al*., 2006).

Secreted virulence proteins of *S. aureus* can be categorized based on proven or putative function. Cytolytic toxins, such as haemolysins (Hla, Hlb, HlgABC) and leukocidins (LukD/E and Panton–Valentine leukocidin, PVL), oligomerize to form pores on the cell surface (Walev *et al*., 1993; Bhakdi *et al*., 1998). Destruction of leucocytes (especially neutrophils), which can be mediated by these toxins, is likely a key component of CA-MRSA pathogenesis. For example, PVL has high specificity for granulocytes and is linked by epidemiology to CA-MRSA disease (Lina *et al*., 1999), although our recent studies indicate the toxin has a limited role in virulence (Voyich *et al*., 2006). Staphylococcal enterotoxins are secreted superantigens (SAg) that bind to major histocompatibility complex (MHC) class II proteins, resulting in CD4+ T-cell activation and immune modulation (Malchiodi *et al*., 1995; McCormick *et al*., 2001; Orwin *et al*., 2002; Llewelyn *et al*., 2004). *S. aureus* also secretes numerous proteases and lipases that degrade host components, and proteins that sequester antibody or inactivate antibiotics (Foster, 2005). As a step towards understanding the role played by *S. aureus* exoproteins in virulence, previous proteomics-based studies identified immunogenic proteins produced by strain COL (Vytvytska *et al*., 2002), evaluated the role played by *S. aureus agr* and *sigB* on secretion of virulence factors (Ziebandt *et al*., 2001), and tested the effects of linezolid on production of virulence factors (Bernardo *et al*., 2004). However, a comprehensive analysis of the exoproteins produced by CA-MRSA has not been conducted.

To that end, we used a proteomic approach to identify exoproteins of LAC and MW2 during growth *in vitro* and evaluated immunogenicity of the proteins using sera from mice infected with each strain. In addition, we used confocal microscopy to determine that selected exoproteins were produced within phagocytic vacuoles of human neutrophils following uptake, a phenomenon accompanied by host cell lysis.

## Results

### Resolution and identification of culture supernatant proteins produced by CA-MRSA strains

**Fig. 1:**
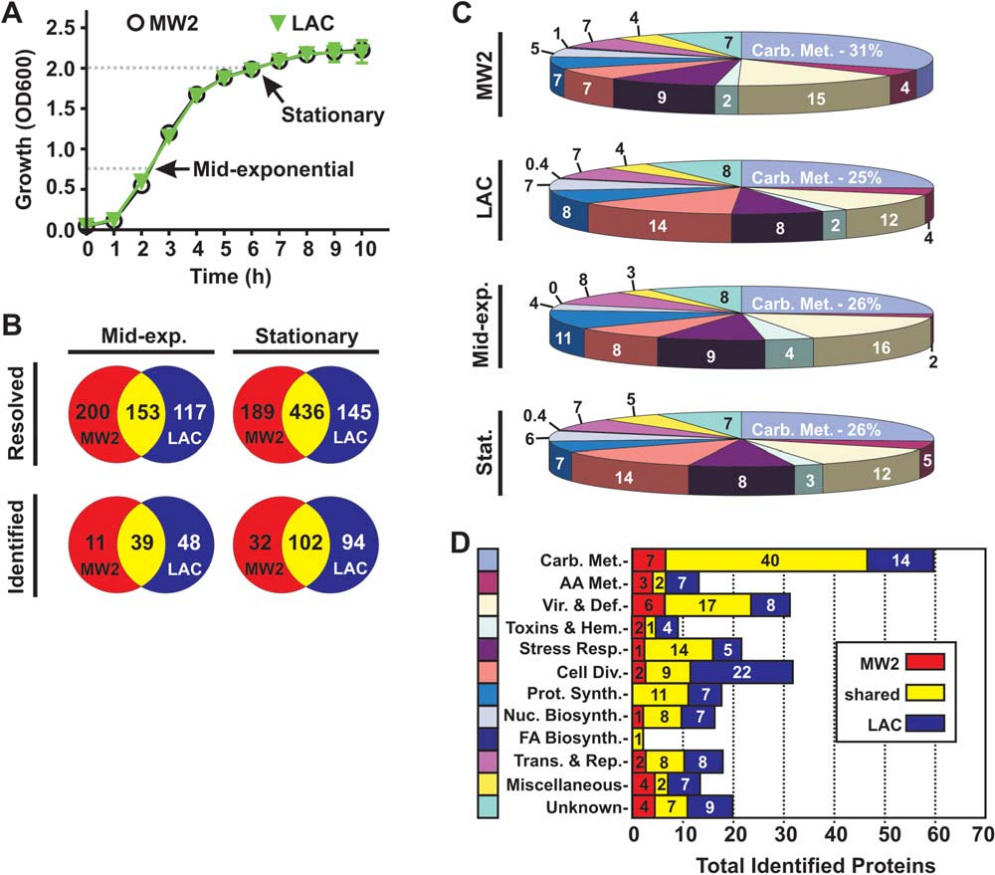
Distribution and function of proteins found in MW2 and LAC culture supernatants. A. MW2 and LAC were cultured to mid-exponential (Mid-exp.) or stationary phases of growth as indicated, at which point proteins were identified/resolved using proteomics as described in *Experimental procedures*. B. Numbers in yellow-shaded regions represent proteins identified in both MW2 and LAC supernatants at the indicated growth phase. Numbers in red- or blue-shaded regions indicate proteins identified in MW2 or LAC supernatants respectively. Results are derived from three separate experiments. C. Proteins identified by ADI-MS/MS were categorized based upon functional annotation. Numbers are the per cent of total proteins identified. D. Proteins identified at mid-exponential and stationary phases of growth were enumerated and categorized by functional annotation. Carb. Met., carbohydrate transport and metabolism; AA Met., amino acid transport and metabolism; Vir. & Def., virulence and defence mechanisms; Toxins & Hem., toxins and haemolysins; Stress Resp., stress response; Cell Div., cell division and maintenance; Prot. Synth., protein synthesis; Nuc. Biosynth., nucleotide biosynthesis; FA Biosynth., fatty acid biosynthesis; Trans. & Rep., transcription and replication.

**Fig. 2:**
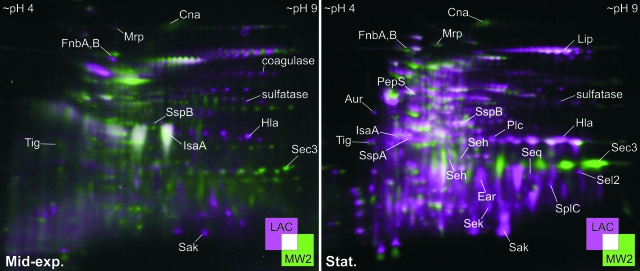
2-DGE analyses of culture supernatant proteins produced by MW2 and LAC. Proteins from MW2 (green) and LAC (magenta) culture supernatants were analysed by proteomics. Left panel, proteins from cultures at mid-exponential (Mid-exp.) phase of growth. Right panel, proteins from cultures at stationary (Stat.) phase of growth. White areas are regions of overlap. Selected proteins are indicated. Images are representative of three separate experiments.

As an initial step towards gaining a comprehensive understanding of exoproteins made by the most prominent CA-MRSA strains, we resolved/identified proteins in MW2 (USA400) and LAC (USA300) culture supernatants using 2-dimensional gel electrophoresis (2-DGE) coupled with automated direct infusion-tandem mass spectrometry (ADI-MS/MS). Three hundred and fifty-three and 270 protein spots were resolved from MW2 and LAC culture media, respectively, at mid-exponential phase of growth (Fig. 1A–D). By comparison, 625 MW2 and 581 LAC proteins were resolved from culture supernatants at stationary phase of growth (Fig. 1B). Of the resolved exoproteins, 153 (60.2 ± 18%) from cultures at mid-exponential growth and 436 (67.9 ± 6.6%) from cultures at stationary growth matched between strains, indicating MW2 and LAC produce numerous proteins that co-migrate during 2-DGE (Fig. 2). We note that this analysis fails to account for variations of same or similar proteins with slightly different migration by 2-DGE. Therefore, our estimate of the degree of similarity between the two strains at the level of exoprotein (60% to 68%) is relatively conservative.

Using ADI-MS/MS and excluding protein isoforms and identifications from multiple gels, we identified 250 unique proteins from MW2 and LAC culture supernatants at the two phases of growth combined (98 at mid-exponential phase of growth and 228 at stationary phase) (Fig. 2 and Table 1). Proteins were separated into categories based on functional annotation to facilitate subsequent analyses (Fig. 1B and C, and Table 1).

**Table 1:** MW2 and LAC culture supernatant proteins identified by ADI-MS/MS.

Protein name (Entrez protein name)^a^	Entrez protein^a^	MW^E^	pI^E^	MP	SC%	MS/MS MOWSE score
Carbohydrate transport and metabolism (61)						
Acetate kinase (AckA)^C1,D1^	13701506	44029	5.7	10	31	226
Acetoin reductase (ButA)^D2^	49482369	27199	5.0	3	15	104
Aconitate hydratase (AcnA)^B1,C1,D1^	49241672	98850	4.9	24	34	878
Adenylate kinase (Adk)^B1,D2^	49484445	23959	4.8	6	29	245
Adenylosuccinate synthetase, putative (PurA)^C1^	49482270	47522	5.1	4	12	146
Alcohol dehydrogenase (Adh)^B5,D6^	14246373	36039	5.5	13	45	558
Alcohol dehydrogenase, zinc-containing (AdhE)^D1^	87162223	36244	5.3	4	13	93
Aldehyde dehydrogenase (AldA1)^B1,D1^	49242475	51936	5.1	7	19	179
ATP synthase beta chain (AtpD)^A1,C2,D1^	49484327	51368	4.7	4	11	101
ATP synthase alpha chain (AtpA)^D1^	49484329	54550	4.9	4	9	82
Citrate synthase II (GltA)^B2,C1,D2^	49483937	42566	5.4	14	50	507
CoA synthetase protein, putative (FadE)^B1,C1,D1^	49483408	42060	4.9	9	23	256
Deoxyribose-phosphate aldolase (Dra)^B1^	14247910	23327	4.7	3	17	56
Dihydrolipoamide acetyltransferase: subunit E2 (PdhC)^A2,C2,D2^	581570	46411	4.9	16	46	817
Dihydrolipoamide dehydrogenase: subunit E3 (PdhC)^A2,B2,C1,D2^	48874	49421	5.0	16	46	803
Phosphoglycerate mutase (Gpm)^B2^	14246544	56419	4.7	12	29	590
Enolase (Eno)^A2,B3,C2,D4^	6015099	47088	4.5	18	54	1257
Methylenetetrahydrofolate dehydrogenase (FolD)^B2,D1^	49244345	30824	5.4	13	68	859
Formate tetrahydrofolate ligase (Fhs)^B1,C1,D1^	83288210	59876	5.8	16	44	759
Formate acetyltransferase (PflB)^D2^	49482458	84808	5.3	6	11	193
Fructose bisphosphate aldolase class I (Fba)^A1,B4,C2,D6^	88196553	33034	4.9	14	57	683
D-Fructose 6-phosphate amidotransferase (GlmS)^D1^	49484376	65839	4.9	6	15	151
6-Phospho-beta-galactosidase (LacG)^A1^	644835	116131	5.3	1	1	42
Glucose 6-phosphate isomerase A (GpiA)^A1,B1,C1,D1^	49244181	49777	4.8	20	53	1008
Glucose 6-phosphate 1-dehydrogenase (Zwf)^D1^	49483755	56929	5.3	7	15	227
Glyceraldehyde 3-phosphate dehydrogenase 1 (Gap)^A2,B5,C3,D5^	49244087	36258	4.9	13	47	770
Glyceraldehyde 3-phosphate dehydrogenase subunit B (GapB)^D1^	38195941	36899	6.0	4	14	106
Glyceraldehyde 3-phosphate dehydrogenase subunit C (GapC)^C1,D1^	38195943	36227	4.9	3	10	65
Glycerate dehydrogenase (MW2224)^B1,D2^	14248079	34681	5.1	10	41	377
Glycine cleavage system H protein (GcvH)^A1,C1,D2^	49241194	14072	4.0	2	30	194
Glycine C-acetyltransferase, similar to (MW0505)^D1^	14246318	41426	5.3	2	5	58
Hexulose 6-phosphate synthase, putative (SgaH)^B1,D1^	87160968	22404	4.6	12	81	656
Hydrolase (HAD superfamily) (MW0575)^B1,D1^	49241218	27962	4.5	4	16	67
Indole-3-pyruvate decarboxylase, putative (IpdC)^D1^	14245955	60490	5.1	1	2	59
Isocitrate dehydrogenase (Idh)^B1,D1^	49244963	46408	4.9	4	10	129
L-Lactate dehydrogenase 1 (Ddh)^B3,D4^	57286685	34562	5.0	10	34	368
Malate:quinone oxidoreductase (Mqo2)^A1,B1,C1,D1^	21205698	55892	6.2	7	17	301
Mannitol-1-phosphate 5-dehydrogenase (MtlD)^B3,D1^	49242510	40801	5.0	10	36	357
2-C-Methyl-D-erythritol 4-phosphate cytidylyltransferase (IspDF)^B1^	49482491	26640	5.4	3	12	77
NAD-dependent dehydrogenase, putative (MW2068)^B2^	49245380	24057	5.0	15	81	643
Oxoglutarate dehydrogenase (Ogdh)^B1,D1^	21204471	105289	5.4	14	22	401
Phosphate acetyltransferase, putative (Pta)^A1,B4,C1,D5^	22212856	20383	4.8	8	44	523
Phosphoenolpyruvate carboxykinase (PckA)^B1,D1^	49484033	59370	5.7	20	44	678
Phosphogluconate dehydrogenase (Pgd)^A1,C1,D1^	14247282	51751	5.1	5	15	122
Phosphopentomutase, putative (Drm)^A1,B1,C2,D2^	14246544	56419	4.7	14	36	672
Phosphoenolpyruvate-protein phosphatase (PtsI)^B1,D1^	1070386	63179	4.7	12	22	490
6-Phosphofructokinase (PfkA)^B2,D1^	49244967	34818	5.6	11	34	327
6-Phosphogluconate dehydrogenase, decarboxylating (Gnd)^B1,D2^	49483761	51770	5.0	12	30	564
Phosphoglycerate kinase (Pgk)^A1,B2,C3,D2^	49483031	42575	5.2	14	47	615
1-Pyrroline-5-carboxylate dehydrogenase (RocA)^B1,D1^	21205647	56833	5.0	16	39	687
Pyruvate carboxylase, putative (PycA)^B1,D1^	49483277	128451	5.2	14	14	353
Pyruvate dehydrogenase E1 component, alpha subunit, putative (PdhA)^A1,C2^	49244374	41357	4.9	7	32	239
Pyruvate dehydrogenase E1 component, beta subunit, putative (PdhB)^A2,B4,C1,D3^	57285889	35194	4.7	10	43	525
Pyruvate kinase (Pyk)^A1,B2,C3,D1^	49242068	63103	5.2	18	38	610
Short chain dehydrogenase MW2249 (MW2249)^D3^	21205420	31769	4.6	4	12	179
Succinyl-CoA synthetase (SucD)^B1^	49244528	31506	5.5	5	25	154
Succinyl-CoA synthetase (SucB)^D3^	49483408	42060	4.9	7	18	246
Tagatose-bisphosphate aldolase, putative (LacD1)^A1,B6^	49245361	30817	5.0	8	40	371
Transaldolase, putative (MW1721)^B4,D2^	14247553	25742	4.8	7	37	273
Transketolase (Tkt)^B1,D1^	57284532	72206	5.0	19	35	703
Triosephosphate isomerase (Tpi)^A1,B3,C2,D3^	49483032	27245	4.8	8	39	477
Amino acid transport and metabolism (12)						
Alanine dehydrogenase 2 (Ald2)^D1^	49244722	40209	5.2	6	18	173
Amidophosphoribosyltransferase precursor, putative (PurF)^B1^	49244352	54363	6.1	12	23	426
Cysteine synthase (CysK)^C2^	82750220	32969	5.4	8	52	273
3-Deoxy-7-phosphoheptulonate synthase (MW1680)^B1,D1^	49483977	40609	5.8	7	24	229
Glucosamine-fructose 6-phosphate aminotransferase (GlmS)^D1^	14247927	65795	4.9	5	11	110
Glutamine synthetase (GlnA)^B1^	1134886	50808	5.1	11	34	400
Imidazolonepropionase (HutI)^D1^	87162411	45011	5.2	5	15	101
Phosphoribosylformylglycinamidine synthase I (PurQ)^B1^	14246838	24541	5.0	3	21	108
Phosphotransferase system enzyme IIA-like protein (SH1484)^D1^	88195154	17949	4.5	4	33	144
SNO glutamine amidotransferase family protein (MW0475)^D1^	49482749	20617	5.7	6	43	211
Thiamine pyrophosphate enzyme, putative (MW0162)^B1,D1^	49240559	60503	5.0	6	13	181
Urocanate hydratase (HutU)^C1^	14248105	60626	5.2	4	12	55
Virulence/defence mechanisms (31)						
Aminopeptidase PepS (PepS)^D1,S^	87161826	46805	4.8	8	19	226
Aureolysin (Aur)^C1,D1,S^	6119705	56281	5.1	3	8	102
Chitinase (MW0945)^D1,S^	49483226	11338	6.6	2	32	75
Coagulase (Coa)^A4,C7,D1,S^	46540	71675	8.4	20	37	920
Collagen adhesin precursor (Cna)^A2,B1,L,M,S^	21205785	132921	5.9	7	7	199
Ear (Ear)^B1,D1,M,S^	21203924	20322	8.6	4	19	115
Esterase\lipase (MW2501)^B1,D2,S^	14248355	30986	4.7	10	52	415
Fibrinogen-binding protein-related (MW1037)^A1,C1,S^	49244435	12171	10.4	3	27	115
FmtB protein (FmtB)^A1,D1^	14247939	263611	4.6	9	4	229
Fibronectin-binding protein A (FnbA)^C3,L,M,S^	87161146	111642	4.6	9	13	254
Fibronectin-binding protein B (FnbB)^A1,B2,C2,L,M,S^	87162339	103492	4.7	9	12	265
IgG-binding protein (Sbi)^A2,S^	49245643	50099	9.4	11	26	330
IgG-binding protein A precursor (Spa)^A9,C7,D4,L,M,S^	83682325	49338	5.7	16	45	666
Lipase (Lip)^C3,D8,S^	1095875	76845	7.1	19	30	947
Lysophospholipase, putative (MW1732)^D1,S^	87162009	31019	5.1	9	53	306
Metallo-beta-lactamase superfamily protein (YycJ)^B1,S^	49483948	25306	5.0	4	24	101
Mrp protein (Mrp)^C1^	5834649	262876	4.6	9	4	250
1-Phosphatidylinositol phosphodiesterase (Plc)^B1,D3,S^	1172527	35213	6.5	13	52	452
Putative sulfatase (MW0681)^B1,C1,D2,M,S^	49244034	74353	9.0	8	17	349
SspA, V8 protease (SspA)^B2,D4,S^	12025238	36304	5.0	6	27	337
SspB, cysteine protease precursor (SspB)^A1,B3,C2,D9,S^	12025239	44491	5.7	15	44	738
Serine protease SplC (SplC)^B1,D2,S^	88195634	26082	6.3	7	27	212
Staphylokinase precursor (Sak)^B4,C1,D6,S^	21205055	18483	6.8	7	67	411
Succinyl-diaminopimelate desuccinylase (MW1943)^B1^	13701801	45109	4.6	6	16	158
Tetracycline resistance protein (TetP)^B1,M,S^	6094458	72677	5.3	1	2	60
ThiJ/PfpI family protein, protease 1 (MW1815)^B3,D2^	49240910	32171	5.0	6	67	323
Trigger factor (prolyl isomerase) (Tig)^A2,B3,C2,D2^	49483918	48565	4.3	8	24	439
Tripeptidase, similar to (MW1465)^B1^	14247283	40172	5.0	4	12	88
Truncated MHC class II analogue protein (SAOUHSC 02466)^D4^	88196118	15438	8.7	7	55	300
Xaa-His dipeptidase (MW1694)^B2,C1,D1,S^	49245019	52775	4.6	13	34	535
Xaa-Pro dipeptidase (MW1482)^B1,D3,S^	49483779	39357	5.2	9	32	314
Toxins and haemolysins (7)						
Alpha-haemolysin, chain G (Hla)^C3,D4,S^	2914575	33227	7.9	14	51	666
Enterotoxin C3 (Sec3)^B4,S^	295149	27634	7.2	17	58	799
Enterotoxin H (Seh)^B4,S^	9955226	25128	5.2	7	38	282
Enterotoxin K (Sek)^C1,D2,S^	87161791	27733	8.3	6	25	252
Enterotoxin L, extracellular (Sel2)^D2,S^	14247781	27479	9.0	2	4	83
Enterotoxin Q (Seq)^B2,C2,D2,S^	87161054	28129	7.7	8	43	358
Exotoxin (SAUSA300_0407)^C1,S^	88194194	25350	8.5	2	15	96
Stress response proteins (20)						
Alkaline shock protein 23 (Asp23)^A1,B7,C5,D11^	49484402	19180	5.1	8	60	301
Alkyl hydroperoxide reductase subunit C (AhpC)^A1,B2,C2,D4^	49482631	20963	4.9	7	53	514
Alkyl hydroperoxide reductase subunit F (AhpF)^B1,D1^	14246148	54674	4.7	5	11	164
ATP-dependent Clp proteinase chain (ClpP)^B2,D3,M^	14248322	77810	4.8	25	49	1416
Catalase (KatA)^B1,C1,D2^	7161887	58287	5.3	15	31	680
Chaperone protein DnaK, HSP70 (DnaK)^A2,B1,C2,D,M^	1169381	66307	4.6	15	34	875
Chaperone protein HchA, Hsp31 (HchA)^B1^	49240910	32171	5.0	4	14	147
Cold shock protein (CspA)^B2,C2,D2^	49483592	7317	4.5	3	78	188
General stress protein 26 (MW2302)^B1,D1^	49245607	15807	5.1	4	40	199
GroES (GroES)^D1^	18028156	10453	5.1	2	26	68
NAD(P)H-flavin oxidoreductase, similar to (Frp)^D1^	14248297	25359	5.5	4	17	56
Nitric oxide dioxygenase (MW0216)^D1^	14246007	42932	5.2	4	12	104
OsmC-like protein (MW0781)^B3,D1^	49244117	15320	4.8	3	25	113
Peroxiredoxin reductase (AhpF)^D1^	49243746	11325	6.4	3	42	67
SrrA (SrrA)^D1^	37781574	28143	5.2	6	30	288
Superoxide dismutase (SodA)^A1,B2,C1,D2^	49483802	22697	5.1	6	45	303
Thioredoxin (TrxA)^B1,D2^	49483308	11433	4.4	5	56	223
Thioredoxin (MW1870)^B1,D1^	49484170	21902	5.2	7	37	259
Thioredoxin reductase (TrxB)^B1,C1,D2^	32468851	33595	5.2	9	30	456
Universal stress protein, putative (MW1653)^B1,D5^	49483951	18463	5.6	8	63	497
Cell division and maintenance (33)						
Aminoglycoside phosphotransferase (AphA)^C1^	11991167	30624	4.5	3	16	64
Autoinducer-2 production protein (LuxS)^D1^	49484358	17502	5.4	4	23	140
Autolysin (Atl)^A2,B1,C3,D4,S^	14248419	69186	6.0	20	43	867
Cyclophilin type peptidyl-prolyl *cis*-*trans* isomerase, putative (MW0836)^D1^	49483114	21605	4.6	4	18	108
Cell division initiation protein DivIVA (MW1335)^B1,D1^	49483601	29963	4.4	3	11	114
Cell division protein FtsZ (FtsZ)^A1,B1,D2^	38604824	41413	5.0	5	15	167
Cytosol aminopeptidase family protein (PepA)^D1^	49483102	54140	5.7	9	23	215
GAF domain protein (MW1661)^D1^	88195528	17100	4.9	3	22	93
HMG-CoA synthase (MvaS)^B3,D1^	9937361	43191	5.0	10	31	414
Histidine-containing phosphocarrier protein (PtsH)^D2^	46908	9505	4.4	2	27	109
Ferritin (Ftn)^B3,D7^	49242263	19590	4.7	6	31	450
Putative non-haem iron-containing ferritin (MW2063)^D2^	49484363	16681	4.6	2	19	120
Formylmethionine deformylase homologue (Def)^D1^	14246861	20546	5.8	6	38	111
Fumarylacetoacetate (FAA) hydrolase family protein (Faa)^B1^	49244187	33093	4.8	3	13	72
Methionine aminopeptidase (Map)^D1^	49484129	27485	5.2	3	14	105
Malonyl CoA-acyl carrier protein transacylase (FabD)^D1^	14247000	33628	4.9	5	23	109
Manganese-dependent inorganic pyrophosphatase (PpaC)^A1,C1,D1^	492245182	34279	4.7	6	23	199
Monofunctional biosynthetic peptidoglycan transglycosylate (MW1814)^D1^	49483860	11941	3.9	2	10	84
Naphthoate synthase (MenB)^D1^	14246815	30392	5.4	6	18	162
Peptidoglycan hydrolase (LytM)^C2,S^	2239274	35147	6.1	4	20	207
Putative 3-methyl-2-oxobutanoate hydroxymethyltransferase (PanB)^D1^	49245819	29237	5.6	8	30	348
Secretory antigen precursor SsaA (SsaA)^D1^	87159889	17388	5.8	1	9	73
Stage V sporulation protein (SpoVG)^B1^	13700388	12312	4.9	3	33	62
tRNA, Arginyl-tRNA synthetase (ArgS)^B1,D1^	49240966	62312	5.1	7	16	220
tRNA, Aspartyl-tRNA synthetase (AspS)^D1^	49483875	66527	5.0	10	17	163
tRNA, Cysteinyl-tRNA synthetase (CysS)^D1^	46395518	53651	5.3	9	23	280
tRNA, Glutamyl-tRNA amidotransferase subunit B (GatB)^C1,D3^	82751553	53607	5.1	9	24	229
tRNA, Glycyl-tRNA synthetase (GlyS)^D1^	49483813	53586	5.0	4	8	107
tRNA, Isoleucyl-tRNA synthetase (IleS)^D1^	49244476	104825	5.3	8	10	179
tRNA, Leucyl-tRNA synthetase (LeuS)^B1,D1^	21204871	91926	5.0	9	17	343
tRNA, Phenylalanyl-tRNA synthetase beta subunit (PheT)^C1,D1^	14246909	88885	4.7	9	13	317
tRNA, Seryl-trna synthetase (SerS)^B1,D1^	14245776	48609	5.0	10	27	327
tRNA, Threonyl-tRNA synthetase 1 (ThrS)^C1,D1^	14247455	74341	5.3	11	20	281
Protein synthesis (18)						
Acetyltransferase (GNAT) family protein (MW2324)^D2^	49483339	16991	4.9	5	32	174
Aminotransferase, putative (RocD)^B1,C1^	492241363	54376	6.1	7	19	229
Branched-chain amino acid aminotransferase (IlvE)^D1^	82750262	40061	5.0	5	20	220
Deblocking aminopeptidase, similar to (MW1253)^D1^	49483560	37848	5.3	1	3	58
Formiminoglutamase (HutG)^D1^	87160628	34491	5.4	6	29	223
Glutamine ammonia ligase (GlnA)^A1,C1,D1^	14247080	50822	5.1	9	34	314
30S Ribosomal protein S1 (RpsA)^A1,B1,C1,D2^	14247247	43283	4.6	15	51	740
30S Ribosomal protein S2 (RspB)^B2,C1,D1^	57286011	29377	5.3	7	26	306
30S Ribosomal protein S6 (RpsF)^A1,B2,C2,D3^	49482595	11588	5.1	6	58	268
S30EA Family ribosomal protein, putative (MW0714)^B1,D2^	49244067	22199	5.2	5	31	211
50S Ribosomal protein L7/L12 (RplL)^A2,B2,C2,D4^	88194302	12704	4.6	7	72	352
50S Ribosomal protein L10 (RplJ)^B1,C1,D1^	49243847	17672	4.8	5	51	242
50S Ribosomal protein L25 (RplY)^C1^	49243808	23773	4.4	4	23	74
O-acetylserine (thiol)-lyase, putative, cysteine synthase (CysK)^B2,C1,D2^	49243820	32955	5.4	11	58	500
Ornithine aminotransferase (RocD1)^B1,D3^	49483117	43444	5.4	11	31	543
Secretory antigen precursor (SsaA)^C3^	49242648	16997	5.8	1	9	86
Secretory antigen precursor SsaA (MW0627)^C1^	49243980	28155	6.1	3	14	69
Serine hydroxymethyltransferase (GlyA)^B1,D1^	49245349	45144	5.8	10	29	422
Nucleotide biosynthesis (16)						
Adenylosuccinate lyase (PurB)^D1^	49484149	49572	5.6	7	18	154
Adenylosuccinate synthase (PurA)^B1,D2^	49482270	47522	5.1	5	14	217
Amidophosphoribosyltransferase precursor (PurF)^B1,D2^	49244352	54363	6.1	12	23	426
Carbamoyl-phosphate synthase large chain (CarB)^C1^	14246973	117098	4.9	3	3	54
GMP synthase (GuaA)^D2^	38372353	58149	4.9	6	13	200
Inositol-monophosphate dehydrogenase (GuaB)^A1,B1,C1,D1^	21203531	52790	5.6	10	35	338
Phosphoribosylamine-glycine ligase (PurD)^D1^	14246844	41946	5.0	2	6	50
Phosphoribosylaminoimidazole-succinocarboxamide synthase (PurC)^B1,D1^	88194764	26676	5.3	12	62	494
Phosphoribosylformylglycinamidine cyclo-ligase (PurM)^D2^	14246841	36966	4.8	7	26	252
Phosphoribosylformylglycinamidine synthetase (PurL)^B1,D1^	14246839	79513	4.8	10	16	245
Phosphoribosylformylglycinamidine synthase (PurS)^D2^	14246838	24541	5.0	7	43	215
Phosphoribosylformylglycinamidine synthase, PurS component (MW0950)^B2^	49241360	9929	4.7	4	68	225
Polyribonucleotide nucleotidyltransferase (PnpA)^B1,D1^	49244556	77342	4.9	14	23	530
Purine nucleoside phosphorylase (DeoD1)^A1,B3,D2^	49484362	25892	4.9	6	35	329
Pyridoxine biosynthesis protein (MW0474)^A1,C1,D1^	49482748	31972	5.1	8	31	267
Uracil phosphoribosyltransferase (Upp)^D1^	49484336	23035	6.1	9	58	496
Fatty Acid Biosynthesis (1)						
Trans-2-enoyl-ACP reductase (FabI)^B1,D1^	56001093	24601	5.2	7	53	226
Transcription and Replication (18)						
2′−5′ RNA ligase (MW0896)^B1,D1^	49244233	19315	4.9	6	40	201
Accessory gene regulator A (AgrA)^D1^	14247812	27903	5.9	5	18	92
DNA-directed RNA polymerase alpha chain (RpoA)^C2^	49484440	34990	4.7	9	40	292
DNA polymerase III, beta chain (DnaN)^C1,D1^	49482255	41888	4.7	8	25	194
DNA-directed RNA polymerase delta subunit (RpoE)^C1^	49245364	20868	3.6	2	14	84
Translation elongation factor G (Fus)^A2,B2,C3,D2^	49243855	76564	4.8	19	40	915
Elongation factor TS (Tsf)^A2,B3,C3,D5^	14247027	32473	5.2	15	57	683
Elongation factor P, putative (Efp)^D1^	49483778	20541	4.8	2	11	82
RsbW (RsbW)^B1^	37781578	17896	4.7	3	20	70
TatD related Dnase, putative (MW0446)^D1^	49482718	29263	5.1	4	17	126
Transcription pleiotropic repressor (CodY)^B1,D2^	49483418	28737	5.9	8	35	354
Transcription termination-anti-termination factor (NusA)^C1,D1^	14247036	43729	4.6	9	29	242
Transcriptional regulator (MW0363)^B2,D3^	14246155	15127	5.0	7	65	301
Transcriptional regulator, LytR family (SAUSA300_0958)^D1^	1723223	23880	5.7	5	33	170
Transcriptional regulator, LytR family (MW0939)^C1,B2^	57284435	45657	6.0	6	20	144
Translation initiation factor IF-1 (InfA)^B1^	49484444	8274	6.7	2	38	64
Translation elongation factor Tu (Tuf)^A2,B1,C2,D3^	49243856	43077	4.7	16	59	871
Transposase (MW2398)^B2,D2^	49484688	16446	5.6	4	33	191
Miscellaneous (13)						
IIIG9 protein, similar to- (LOC576703)^B1^	72179405	52711	9.4	1	2	51
6,7-Dimethyl-8-ribityllumazine synthase (RibH)^B1,D1^	49242141	16412	5.7	7	73	342
ABC transporter-associated protein, SufB (MW0799)^D1^	49483078	52512	5.1	7	18	139
Amylase (MalA)^D1^	18145251	77435	5.9	1	1	54
Aldo/keto reductase family protein (MW2127)^D1^	49482959	32339	5.2	4	12	114
Arsenate reductase family protein (MW0785)^D1^	49483064	13591	6.7	3	49	133
Lipoate synthase (LipA)*^A1^	27807337	16468	6.3	2	17	54
Cell wall surface anchor family protein (MW2416)^C1,D1^	57285190	136262	5.7	16	16	804
Short chain dehydrogenase (MW2249)^B1^	14248102	31777	4.7	2	5	72
Immunodominant antigen A (IsaA)^A9,B5,C13,D2^	14248343	24189	5.9	4	23	259
N-Acetylglucosamine 6-phosphate deacetylase (NagA)^D1^	87161324	43089	5.4	7	20	123
4-Nitrophenylphosphatase-probable (MW0811)^B1^	82750544	27962	4.5	4	16	61
SufD (SufD)^D1^	82750525	48518	5.4	5	18	69
Unknown (20)						
Conserved hypothetical protein (SAUSA300_0871)^D1^	88194663	33093	4.8	9	42	262
Conserved hypothetical protein (SAUSA300_0916)^D1^	88194708	19314	5.0	7	47	249
Conserved hypothetical protein (SAUSA300_1856)^D2^	88195657	19536	6.1	7	46	247
Hypothetical exported protein (MW0347)^B6,D7^	49243694	21261	5.7	3	14	115
Hypothetical exported protein (MW2606)^B1,D1^	82752265	18700	4.7	3	25	147
Hypothetical cytosolic protein (MW0395)^C1,S^	14246202	55465	5.1	14	32	487
Hypothetical protein (MW0542)^B1^	14246355	29371	5.1	3	14	97
Hypothetical protein (MW0819)^B1,D1,M^	49244219	69762	5.1	14	30	604
Hypothetical protein (MW2068)^D1,S^	4126674	22954	5.2	10	62	369
Hypothetical protein (SAUSA300_0408)^A1,C5,S^	57285506	56443	4.8	19	40	899
Hypothetical protein (SAUSA300_0279)**^D1,M,S^	77383233	24390	6.9	1	5	51
Hypothetical protein (MW1884)^A1,M,S^	30043928	13044	9.3	2	26	99
Hypothetical protein (MW0577)^B1,S^	49482843	18554	9.2	4	25	124
Hypothetical cytosolic protein (MW1786)^B1,C1,D1^	49484087	13302	4.4	9	85	406
Hypothetical protein (MW1795)^A2,B1,D2^	49484096	22344	5.3	10	58	394
Hypothetical protein (MW2099)^B1,D1^	49484393	10000	6.1	3	45	95
Hypothetical protein (SAUSA300 2327)^D1^	87161861	15876	4.9	7	58	336
Hypothetical protein (SAUSA300 pUSA010004)^C1,M,S^	87159841	21257	9.3	2	11	55
Putative exported protein (MW0355)^B8,M^	49243745	56170	5.0	17	45	620
Putative exported protein (MW1757)^D2,S^	49245076	20371	6.8	3	21	100

a The Mascot search results displayed above (Entrez Protein number, MW, pI, MP, SC%, and MS/MS MOWSE score) are from the best protein match to published *S. aureus* genomes. In many instances, database searches were performed before the genome sequence of USA300 was published and the best match is to a protein from another published *S. aureus* strain. However, the Entrez Protein name indicates the name of the likely MW2 or USA300 protein. SC, sequence coverage. MP, matched peptides.

^A^MW2 supernatants from mid-exponential phase of growth.

^B^MW2 supernatants from stationary phase of growth.

^C^LAC supernatants from mid-exponential phase of growth.

^D^LAC supernatants from stationary phase of growth.

^E^Theoretical or predicted.

^L^Contains an LPXTG cell wall anchoring signal sequence.

^M^Contains probable transmembrane regions.

^S^Contains a probable N-terminal signal peptide sequence.

^1–8^Number after the letter ^A,B,C^ or ^D^ indicates number of times a protein was identified.

* Best Mascot search homology was to Cathelicidin 4 [indolicidin] [*Bos taurus*].

** Best Mascot search homology was to Pfl_3008 [*Pseudomonas fluorescens PfO-1*] (ABA74746).

### Identification of CA-MRSA exoproteins associated with virulence

Twenty exoproteins (20%) identified at mid-exponential growth and 33 (15%) of those at early stationary phase of growth are known to be associated with virulence (Fig. 1 and Table 1). There were essentially three subcategories of virulence determinants found in culture media. Proteases or enzymes, including aminopeptidase (PepS), aureolysin (Aur), staphylokinase (Sak), V8 protease (SspA), cysteine protease (SspB), serine protease (SplC), lipase (Lip) and Xaa-Pro dipeptidase, were produced by each strain at either phase of growth (Fig. 1 and Table 1). These enzymes degrade and/or modify proteins and lipids present in the growth environment (McGavin *et al*., 1997; Karlsson *et al*., 2001; Massimi *et al*., 2002; Imamura *et al*., 2005). For example, cysteine protease/staphopain B (SspB), which directly cleaves kininogen, also works in concert with staphopain A (ScpA) to promote vascular leakage and lower blood pressure, thereby facilitating septic shock (Massimi *et al*., 2002; Imamura *et al*., 2005). SspA causes release of cell surface fibronectin-binding protein (FnbB) and immunoglobulin G (IgG)-binding protein A (protein A, Spa), modifying capacity for host interaction and increasing free FnbB and Spa (McGavin *et al*., 1997; Karlsson *et al*., 2001).

A second group of molecules identified in CA-MRSA culture media were those involved in bacteria–host interaction or adhesion, such as coagulase (Coa), collagen adhesin (Cna), enolase (Eno), fibrinogen-binding protein, FnbA, FnbB, 1-phosphatidylinositol phosphodiesterase (Plc), Spa and IgG-binding protein (Sbi) (Figs 1 and 2, and Table 1). These molecules can activate the clotting cascade (Coa) (Panizzi *et al*., 2006), mediate binding to host tissues (Cna, Eno, FnbA and FnbB) (Patti *et al*., 1992; Greene *et al*., 1995; Carneiro *et al*., 2004), and sequester host antibody (Spa and Sbi) (Forsgren and Sjoquist, 1966; Zhang *et al*., 1998). Although the function of *S. aureus* Plc is uncharacterized, that of *Listeria monocytogenes* promotes adhesion to epithelial cells and mediates escape of the pathogen from phagosomes (Krawczyk-Balska and Bielecki, 2005; Wei *et al*., 2005).

The toxins and haemolysins, namely alpha-haemolysin (Hla), enterotoxin C3 (Sec3), enterotoxin H (Seh), enterotoxin K (Sek), enterotoxin L (Sel2) and enterotoxin Q (Seq), comprised at most 4% of the exoproteins produced by MW2 and/or LAC in mid-exponential or early stationary phases of growth (4/98 at mid-exponential phase and 6/229 at early stationary phase of growth respectively) (Fig. 1 and Table 1). Unexpectedly, we failed to detect gamma-haemolysin subunits (HlgA, HlgB and HlgC), LukD/E, LukM, or either component of PVL (LukS-PV and LukF-PV) in culture supernatants under the two growth conditions tested.

### Proteins involved in metabolism, biosynthesis, transcription and replication are present in MW2 and LAC culture supernatants

CA-MRSA culture supernatants contained 73 proteins known to be involved in the transport and utilization of carbohydrates or amino acids for energy (Table 1). Thirty-five proteins known to participate in biosynthesis of nucleotides, proteins and fatty acids, and 51 proteins involved in cell division, transcription and replication, were also identified in culture media (Fig. 1 and Table 1). The observation that cytoplasmic proteins were found in culture supernatant is not unexpected, as numerous cycles of autolysis would have occurred thereby releasing proteins into culture medium (Lei *et al*., 2000; Chaussee *et al*., 2001; Trost *et al*., 2005).

### Stress response proteins are present in culture supernatants

Twenty proteins associated with the response to environmental stress were identified in MW2 and LAC culture supernatants (Fig. 1 and Table 1). For example, alkyl hydroperoxide reductase (AhpC and AhpF), catalase (KatA), superoxide dismutase (SodA), thioredoxin (TrxA) and thioredoxin reductase (TrxB), proteins that function to inactivate reactive oxygen species, and heat shock proteins, GroES and DnaK, were identified in culture supernatants at both phases of growth (Fig. 1 and Table 1). Consistent with this observation, genes encoding these proteins are induced in MW2 and LAC during phagocytosis by human neutrophils (Voyich *et al*., 2005). Further, heat shock proteins such as DnaK and GroEL have been shown to be immunogenic in patients with *S. aureus* endocarditis (Qoronfleh *et al*., 1993; 1998), suggesting they are exoproteins *in vivo*.

### MW2 and LAC produced numerous exoproteins of unknown function

We identified 20 culture supernatant proteins with no characterized function, including four putative exported proteins (Fig. 1, Table 1 and Table S1) (Baba *et al*., 2002; Diep *et al*., 2006). Fourteen of these proteins were conserved across 10 sequenced strains of *S. aureus* (Table S1). Genes encoding MW0395 and MW1757 reside within Type II genomic islands of MW2 known as νSaα and νSaβ respectively, and each is located near or among putative virulence determinants (Baba *et al*., 2002). MW1884 is encoded by MW2 prophage ΦSa3 and is juxtaposed to the gene encoding staphylokinase (*sak*), a known virulence factor in *S. aureus* (Baba *et al*., 2002). Several other exoproteins with no characterized function, such as MW0542, MW1795, MW2068, SAUSA300_0871, SAUSA300_0916 and SAUSA300_2327, have homology to enzymes that participate in metabolism or replication, or respond to stress (Table S1). It will be important to determine whether some of these proteins have a role in virulence and/or if they are potential vaccine targets.

### Selected MW2 and LAC exoproteins differ in abundance

**Fig. 3:**
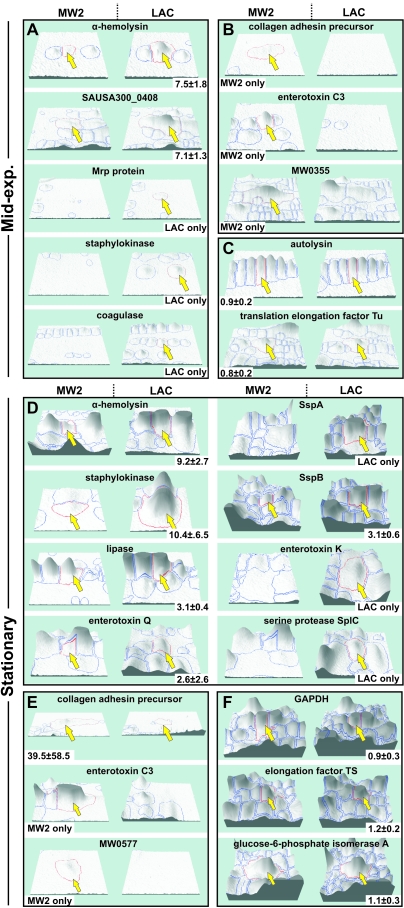
Quantitative analysis of CA-MRSA culture supernatant proteins produced during growth *in vitro*. Differential analysis of MW2 and LAC culture supernatant proteins was performed as described under *Experimental procedures*. Proteins more abundant in/found only in LAC (A and D) or MW2 (B and E) supernatants. Panels C and F represent proteins equally abundant in MW2 and LAC. The phase of growth at which the analysis was performed is indicted to the left of the panels. Results are the mean ± SD of three separate experiments at each phase of growth.

**Fig. 4:**
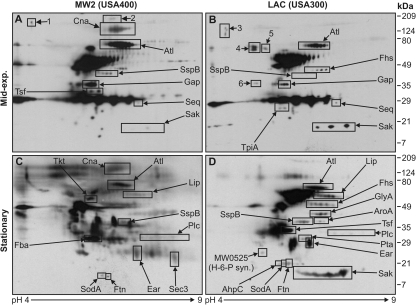
Exoproteins produced by MW2 and LAC during infection. Culture supernatant proteins made by MW2 or LAC at the indicated phase of growth were separated by 2-DGE, transferred to nitrocellulose membranes, and probed with convalescent sera from mice infected with each strain or non-immune sera (not shown). Proteins immunoreactive only with sera from infected mice are indicated (boxed). Unidentified immunoreactive proteins are annotated with Arabic numbers. Immunoblots are representative of three separate experiments.

Although MW2 and LAC produced many common exoproteins, 11 exoproteins detected at either phase of growth differed in abundance between the strains (Figs 3 and 4). Hla and a putative surface protein (SAUSA300_0408) were in greater abundance in LAC culture supernatants at mid-exponential growth phase, and multiple repeat polypeptide (Mrp), Sak, and Coa were found exclusively in the same supernatants (Fig. 3A). By comparison, Cna, Sec3 and a putative exported protein (MW0355) were identified as exoproteins only in MW2 culture supernatants at mid-exponential growth (Fig. 3B).

At stationary phase of growth, Hla, Sak, Lip, Seq, SplC, SspA, SspB and Sek were either increased ≥ 2-fold in LAC supernatants or were found only in those supernatants (Fig. 3D). There was far more Cna (39.5-fold) in MW2 supernatants at this phase of growth, and Sec3 and MW0577, a protein of unknown function, were detected only in MW2 culture media (Fig. 3E). Differences in exoproteins between these strains may underlie in part the noted variances in disease phenotypes (Baba *et al*., 2002; Kazakova *et al*., 2005; Miller *et al*., 2005; Voyich *et al*., 2005; Diep *et al*., 2006).

### Exoproteins made during infection *in vivo*

To reconcile exoproteins produced by MW2 and LAC *in vitro* and those made during infection *in vivo*, we used a mouse abscess model to generate immune/convalescent sera from mice infected with MW2 and LAC (Voyich *et al*., 2006). Following 2-DGE and transfer to nitrocellulose, MW2 and LAC exoproteins were probed with convalescent serum from mice infected with either MW2 or LAC. Several proteins from these CA-MRSA strains were commonly immunogenic in mice (Fig. 4). For example, AhpC, Atl, formate tetrahydrofolate ligase (Fhs), glyceraldehyde 3-phosphate dehydrogenase (Gap), Lip and SspB were immunogenic in mice infected with either strain (Fig. 4 and Table 2). Cna, Sec3 and Sak are known virulence factors that were immunogenic in mice infected with either MW2 (Cna and Sec3) or LAC (Sak) (Fig. 4 and Table 2), findings consistent with the differential analysis of exoproteins produced *in vitro* (compare Figs 3 and 4). Although many other immunogenic proteins were detected by our analysis, many were cross-reactive with non-immune sera (unboxed, unmarked protein spots, Fig. 4) or could not be identified with absolute certainty (indicated by numbers, Fig. 4). Taken together, these data provide strong support to the idea that proven or putative virulence factors, such as Atl, Cna, Lip, Sak, Sec3 and SspB, are made during CA-MRSA infection *in vivo*.

**Table 2:** Immunogenic (*in vivo* expressed) exoproteins of MW2 and LAC.

Protein^a^	MW2	LAC	Immunoreactivity
AhpC, alkyl hydroperoxide reductase	ME, S	ME, S	NI, I
AroA, chorismate mutase	–	S	I
Asp23, alkaline shock protein 23	–	S	NI, I
Atl, autolysin	ME, S	ME, S	I
ClpP, ATP-dependent Clp protease	S	S	NI, I
Cna, collagen adhesin precursor	ME, S	–	I
Coa, coagulase	ME	–	NI, I
DeoD, purine nucleoside phosphorylase	ME	ME	NI, I
Ear	S	S	I
Eno, enolase	S	S	NI, I
Fba, fructose bisphosphate aldolase	S	–	NI, I
Fhs, formate tetrahydrofolate ligase	ME, S	ME, S	I
Ftn, ferritin	–	S	I
Gap, glyceraldehyde 3-phosphate dehydrogenase	ME, S	ME, S	I
GlyA, serine hydroxymethyltransferase	–	S	I
Gpi, glucose 6-phosphate isomerase	ME	ME	NI, I
GuaB, inositol-monophosphate dehydrogenase	ME	–	NI, I
Idh1, isocitrate dehydrogenase	–	S	NI, I
Lip, lipase	S	S	I
MW0525, hexulose 6-phosphate synthase	–	S	I
MW0896, 2′−5′ RNA ligase	–	S	NI, I
MW1795, hypothetical protein	–	S	NI, I
MW1870, thioredoxin	–	S	NI, I
PdhA, pyruvate dehydrogenase subunit A	ME	ME	NI, I
PdhB, pyruvate dehydrogenase subunit B	S	S	NI, I
Pgd, phosphogluconate dehydrogenase	ME	ME	NI, I
Pgi, phosphoglucose isomerase	S	S	NI, I
Plc, 1-phosphatidylinositol phosphodiesterase	S	S	I
Pta, phosphate acetyltransferase	–	S	I
RocD, ornithine aminotransferase	S	–	NI, I
Sak, staphylokinase	ME	ME, S	I
Sec3, staphylococcal enterotoxin C3	S	–	I
Seq, staphylococcal enterotoxin Q	ME	ME	I
SodA, superoxide dismutase	S	S	I
Spa, immunoglobulin-binding protein A	ME, S	ME, S	NI, I
SspB, cysteine protease precursor	ME, S	ME, S	I
Sulfatase	S	–	NI, I
Tkt, transketolase	S	–	I
TpiA, triosephosphate isomerase	–	ME	I
Tsf, translation elongation factor Ts	ME	S	I
Tuf, translation elongation factor Tu	ME, S	ME, S	NI, I
Unknown 1	ME	–	I
Unknown 2	ME	–	I
Unknown 3	–	ME	I
Unknown 4	–	ME	I
Unknown 5	–	ME	I
Unknown 6	–	ME	I

a Protein identities were obtained by overlay analysis as described under *Experimental procedures*. Results are representative of three experiments using serum pooled from 10 to 15 mice.

ME, mid-exponential phase of growth; S, stationary phase of growth; NI, non-immune sera; I, immune sera.

### Phagocytosis of CA-MRSA by human neutrophils triggers production/secretion of virulence factors

**Fig. 5:**
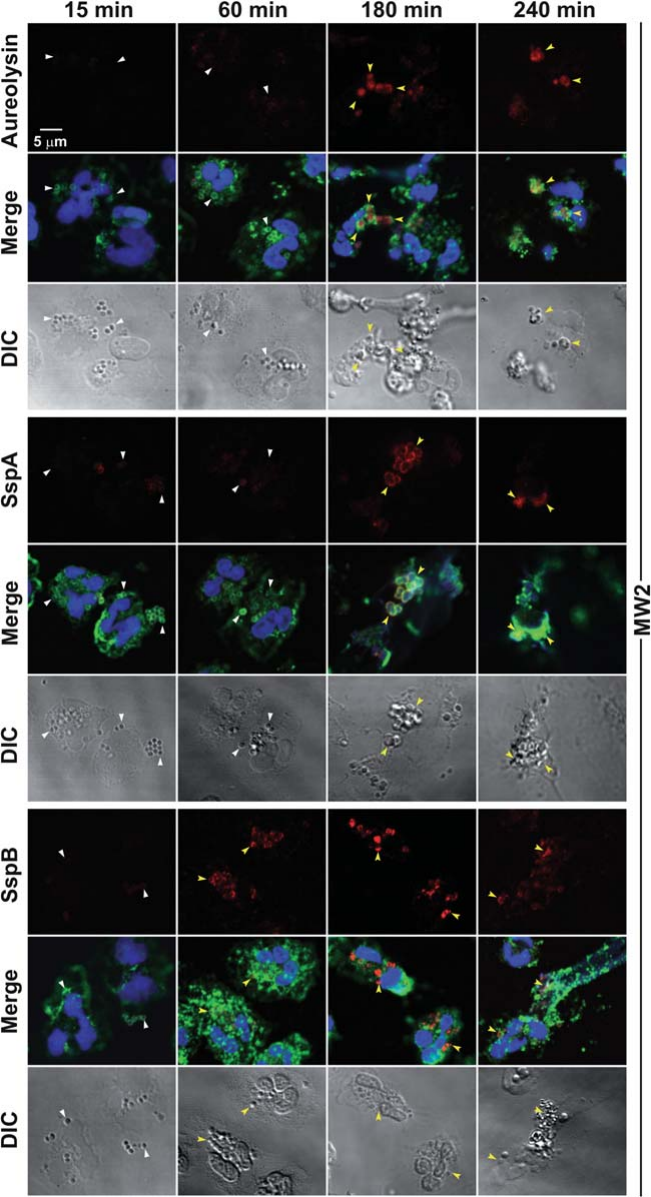
Production and distribution of selected MW2 (USA400) virulence factors during phagocytosis by human PMNs. Following phagocytosis of MW2, aureolysin (Aur), SspA and SspB were visualized by confocal laser-scanning microscopy. White arrowheads indicate bacteria. Yellow arrowheads indicate areas enriched with the *S. aureus* protein of interest. The image labelled ‘Merge’ illustrates distribution of neutrophil actin-related protein (ARP, green) and nuclei (blue). DIC, differential interference contrast.

**Fig. 6:**
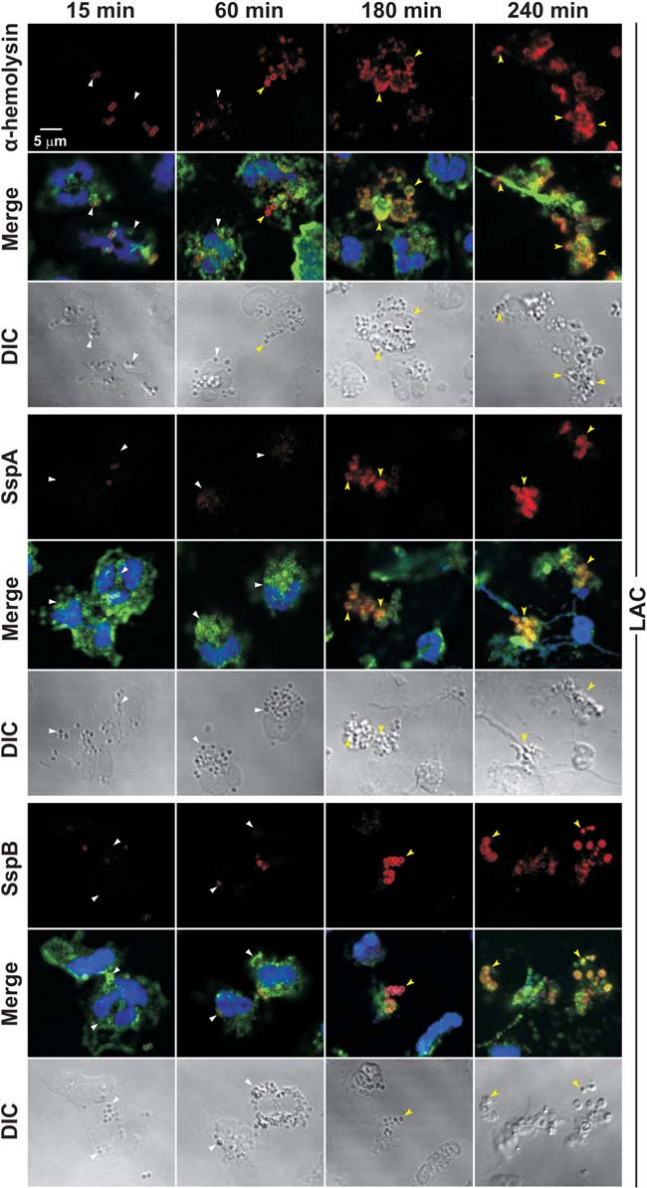
Production and distribution of selected LAC (USA300) virulence factors during phagocytosis by human PMNs. Following phagocytosis of LAC, Hla, SspA and SspB were visualized by confocal laser-scanning microscopy. Labelling for this figure is otherwise identical to the legend for Fig. 5.

To determine if production of selected exoproteins is triggered by interaction of *S. aureus* with host cells and/or if the proteins are made within phagosomes, we used confocal laser-scanning microscopy to evaluate production of Aur, Hla, SspA and SspB after phagocytosis by human polymorphonuclear leucocytes (PMNs) (Figs 5 and 6). Notably, there was a time-dependent increase in Aur, Hla, SspA and SspB produced by MW2 and/or LAC within neutrophil phagocytic vacuoles (Figs 5 and 6, yellow arrowheads). There was also redistribution of each molecule over time; at 15 or 60 min proteins were typically localized only to *S. aureus*, whereas at 3 or 4 h after phagocytosis each molecule was diffused within larger, more mature phagosomes or distributed throughout the cell (Figs 5 and 6, yellow arrowheads). Accumulation of these molecules late during phagocytosis correlates well with the noted PMN lysis caused by MW2 and LAC (Figs 5 and 6) (Voyich *et al*., 2005). Production of virulence factors within phagosomes is consistent with the notion that these proteins are made during infection *in vivo*.

## Discussion

The striking increase in CA-MRSA infections over the past few years has prompted an intense search for the underlying molecular determinants. To date, few virulence factors are associated specifically with CA-MRSA disease and no single determinant appears to account for the increased incidence and severity of CA-MRSA infections (Lina *et al*., 1999; Baba *et al*., 2002; de Bentzmann *et al*., 2004; Diep *et al*., 2004; 2006; Fridkin *et al*., 2005; Voyich *et al*., 2006). It is almost certain that a combination of virulence determinants, including *S. aureus* exoproteins, and host susceptibility promote disease in otherwise healthy subjects. Inasmuch as exoproteins produced by CA-MRSA probably facilitate evasion of innate host defence (Voyich *et al*., 2005) and thereby contribute to disease, we performed a comprehensive analysis of exoproteins produced by MW2 and LAC *in vitro* and during infection.

A limited number of proteomics-based studies have investigated exoproteins of *S. aureus*, typically using strain COL or laboratory-derived strains (Ziebandt *et al*., 2001; 2004; Nakano *et al*., 2002). For example, Ziebrandt *et al*. recently compared *S. aureus* strains RN6390 and RN6911 and identified 43 exoproteins produced *in vitro*, including many controlled by accessory gene regulator (*agr*) and/or alternative sigma factor ó^B^ (*sigB*) (Ziebandt *et al*., 2004). Nakano *et al*. identified 29 exoproteins produced by MRSA strains using 2-DGE coupled with N-terminal peptide sequencing (Nakano *et al*., 2002). By comparison, numerous studies have reported individual *S. aureus* exoproteins that promote pathogenesis, including proteases (McGavin *et al*., 1997; Karlsson *et al*., 2001; Imamura *et al*., 2005), enterotoxins and exotoxins (Dinges *et al*., 2000; McCormick *et al*., 2001), and leukotoxins and haemolysins (Kaneko and Kamio, 2004). Although progress has been made towards identification and characterization of many important *S. aureus* exoproteins, there is a noted paucity of information regarding exoproteins produced by CA-MRSA strains.

Our analysis of MW2 (USA400) and LAC (USA300) culture supernatants identified 250 exoproteins (out of 600+ resolved protein spots) between two phases of growth *in vitro*, at present the single most comprehensive view of *S. aureus* exoproteins. Differential analysis of MW2 and LAC exoproteins revealed key differences between the strains (Fig. 3). These differences were not due to differences in rate of growth between the strains, because MW2 and LAC have essentially identical growth curves *in vitro* (Fig. 1A). Although many of the differentially abundant exoproteins, including Atl, Coa, Hla, Lip, Mrp, Sak, Sek, Seq, Sec3, SspA, SspB and SplC, are relatively ubiquitous among *S. aureus*, it is possible that the observed variances in protein levels relate to distinct strain pathologies. For example, Cna is linked to necrotizing pneumonia (de Bentzmann *et al*., 2004) and there were higher levels of this exoprotein in MW2 culture supernatants (Fig. 3). MW2 is a strain known to cause lethal pneumonia (Centers for Disease Control and Prevention, 1999). Compared with MW2, more Hla was present in LAC culture supernatants (7.5 ± 1.8- and 9.2 ± 1.8-fold more Hla at mid-exponential and stationary phases of growth respectively). Consistent with that observation, Hla appeared to accumulate more rapidly in LAC-containing neutrophil phagosomes or was more highly diffused in and around deteriorating PMNs after phagocytosis of LAC compared with MW2 containing cells (accumulation of Hla typically occurred by 180 min in LAC-containing PMNs versus 240 min in those with MW2). We recently found dramatic differences in pathophysiology between MW2 and LAC in a mouse skin infection/abscess model (Voyich *et al*., 2006). LAC produced rapid and pronounced dermonecrosis in infected animals, whereas mice infected with MW2 developed intact abscesses (Voyich *et al*., 2006). Thus, differences in exoprotein abundance, such as that for Hla, may underlie the differences in strain pathology. Additional studies are needed to test this hypothesis.

We used sera from mice infected with MW2 or LAC to identify exoproteins made during infection *in vivo* (Fig. 4). Previous serological proteome studies using strain COL identified 15 immunogenic proteins made during human infections, although only four of these proteins (alkaline shock protein, hexose 6-phosphate synthase, PdhB and Tuf) are common with our analysis (Table 2) (Vytvytska *et al*., 2002). In more recent work, Clarke *et al*. used bacteriophage expression libraries to identify *S. aureus* antigens produced during human infections (Clarke *et al*., 2006). Several of those immunogenic proteins, i.e. chorismate mutase (AroA), autolysin (Atl), Coa, Fhs, Gap, transketolase (Tkt) and triosephosphate isomerase (Tpi), were also identified as *in vivo* expressed *S. aureus* antigens by our studies (Table 2). Our work revealed many additional exoproteins produced during infection, such as AhpC, Cna, Ear, ferritin (Ftn), Lip, Plc, phosphate acetyltransferase (Pta), Sak, Sec3, Seq and SspB (Fig. 4). Importantly, these proteins were immunoreactive only with sera from MW2- or LAC-infected animals (as opposed to sera from uninfected animals). Variances in antigenic exoproteins between MW2 and LAC are likely explained in part by differences in gene content or phase of growth used to test antigenicity (Fig. 4). The relative importance of these *in vivo* expressed proteins remains to be determined.

At least four of the exoproteins identified by our proteomic analysis (Aur, Hla, SspA and SspB) were produced within phagosomes of human neutrophils following uptake (phagocytosis) (Figs 5 and 6). These findings are notable because MW2 and LAC are known to cause rapid lysis of PMNs (Voyich *et al*., 2005) and the factors responsible for the dramatic host cell lysis remain elusive (Voyich *et al*., 2006). Consistent with these data, we determined previously that the gene encoding Hla was upregulated during phagocytosis (Voyich *et al*., 2005). Although our studies do not demonstrate that Aur, Hla, SspA and SspB are directly related to the observed PMN lysis, increased accumulation of these virulence determinants accompanied initial stages of neutrophil destruction (Figs 5 and 6).

The *S. aureus* genome consists of ∼2600 proteins of which more than 40% have no similarity to proteins of known function (Kuroda *et al*., 2001). Moreover, 33% of identified proteins are unique to *S. aureus* (Kuroda *et al*., 2001). Therefore, it is not surprising that 8.5% of the proteins identified in our study have no known function (Fig. 1). Some of these exoproteins are of significant interest based upon homology to known *S. aureus* proteins (e.g. SAUSA300_0407 as an exotoxin homologue) or given their location within the genome (Table 1 and Table S1). Several exoproteins identified by our analysis are putative exported or surface proteins, e.g. SAUSA300_0408 and MW0355 (rather than metabolism enzymes, etc.) and also require characterization in the context of CA-MRSA pathogenesis (Fig. 3).

We used a proteomics-based approach to generate a comprehensive view of exoproteins made by prominent CA-MRSA strains, including identification of proteins that are immunogenic and thus produced during infection *in vivo*. Identification of these exoproteins is an important first step towards development of vaccines, prophylactics, and enhanced therapeutics designed to control CA-MRSA infections.

## Experimental procedures

### Growth of *S. aureus* and generation of culture supernatants

*Staphylococcus aureus* strains MW2 (USA400) (Baba *et al*., 2002) and LAC (USA300-0114) (Kazakova *et al*., 2005; Miller *et al*., 2005; Voyich *et al*., 2005; Diep *et al*., 2006) were cultured in tryptic soy broth containing 0.25% dextrose (TSB, Becton, Dickenson, and Company, Franklin Lakes, NJ) filtered sequentially through 10 kDa cut-off and 0.22 μm filters. Cultures were inoculated with a 1:200 dilution of overnight culture (500 μl of culture into 100 ml of TSB in a 1 l flask) and incubated at 37°C with shaking (250 rpm). All strains were cultured to mid-exponential (OD_600_ = 0.75) or stationary (OD_600_ = 2.0) phases of growth and placed on ice until used. Bacteria were removed from culture media by two sequential rounds of centrifugation at 2851 *g* for 10 min at 4°C. This procedure yielded clarified culture supernatants for subsequent proteomic analyses.

### Precipitation and preparation of culture supernatant proteins

Precipitation of proteins from clarified culture supernatants was performed in polypropylene flasks to reduce protein loss. One hundred millilitres of clarified supernatant was added to 300 ml of 100% ethanol (Molecular Grade, Sigma-Aldrich, St Louis, MO) and chilled at −20°C overnight. Precipitated protein was transferred to Oakridge centrifuge tubes and sedimented by centrifugation at 27 216 *g* for 30 min at 4°C. To optimize yield, protein in polypropylene flasks (residual) and centrifuge tubes was air-dried for approximately 1 h. Protein in the flasks were solubilized with 3 ml of 2-D solubilization solution (7 M urea, 2 M thiourea, 4% CHAPS) with gentle swirling. These samples were transferred to Oakridge tubes containing precipitated protein pellets and swirled gently to dissolve pellets. Polypropylene flasks were rinsed with an additional 600 μl of 2-D solubilization solution.

Culture supernatant proteins were clarified further with a second precipitation in 30% trifluoroacetic acid (Sigma-Aldrich) and incubated on ice for a minimum of 30 min. Precipitated proteins were collected by centrifugation at 14 100 *g* for 5 min at room temperature. The protein pellet was dispersed by vortexing in 25 μl of distilled H_2_O for 10 s followed by the addition of 1 ml of acetone (−20°C). Proteins were washed in acetone for a minimum of 30 min at −20°C and then pelleted by centrifugation at 14 100 *g* for 5 min at room temperature. Proteins were air dried for ∼1 h or until pellets appeared dry. Pellets were solubilized in 400 μl of Destreak Rehydration Solution (GE Healthcare, Piscataway, NJ) containing tributylphosphine (200 mM) and ampholytes (pH 3–10, 4 μl of 100× solution) (Bio-Rad, Hercules, CA) at room temperature for 1 h with constant swirling. Samples were used immediately or stored briefly at −20°C.

### Isolation of human neutrophils

Human polymorphonuclear leucocytes were isolated from fresh venous blood of healthy individuals using a published method (Kobayashi *et al*., 2002; Burlak *et al*., 2006). Studies were performed in accordance with a protocol approved by the Institutional Review Board for Human Subjects, National Institute of Allergy and Infectious Diseases. PMN preparations typically contained ∼94% neutrophils, with the remaining cells being predominantly eosinophils. All reagents used contained < 25.0 pg ml^−1^ endotoxin (Limulus Amebocyte Lysate assay, Fisher Scientific, Suwannee, GA).

### Neutrophil phagocytosis

MW2 and LAC were cultured to mid-exponential phase of growth and phagocytosis experiments were performed with serum-opsonized bacteria as described (Kobayashi *et al*., 2003; Voyich *et al*., 2005). At the indicated times, phagocytosis was terminated either by chilling PMNs on ice or adding cold paraformaldehyde (4%) to assay wells.

### Generation of immune sera

Female Crl:SKH1-hrBR mice (Charles River Laboratories, Wilmington, MA) were anaesthetized with isoflurane and inoculated with 50 μl of DPBS containing 10^7^ cfu of MW2 or LAC by subcutaneous injection in the right flank. Abscesses typically formed within 4 days and resolved 14 days after infection (Voyich *et al*., 2006). At 28 days post infection, mice were euthanized and blood was collected from 10 to 15 mice to prepare pooled immune serum. Blood from uninfected animals was processed in parallel (non-immune serum). All studies conformed to guidelines set forth by the National Institutes of Health and were reviewed and approved by the Animal Use Committee at Rocky Mountain Laboratories, NIAID.

### Isoelectric focusing and second-dimension SDS-PAGE

Culture supernatant proteins were precipitated in cold acetone and then solubilized with isoelectric focusing (IEF) buffer (7% urea, 2% thiourea, 4% CHAPS and 200 mM tributylphosphine) as described (Burlak *et al*., 2006). Protein concentration was measured with the 2-D Quant kit (GE Healthcare) and purified proteins were stored at −80°C.

For IEF, samples were treated with Destreak Rehydration solution (25% of total sample volume) (GE Healthcare), 200 mM tributylphosphine and 1% ampholytes. IEF was performed with 11 cm IPG Ready Strips for 40 kVh. IPG Ready Strips were rehydrated actively at 50 V overnight prior to first dimension separation. Moistened filter paper wicks (Whatman no. 1 paper, Whatman, Florham Park, NJ) were added between each electrode and strip prior to focusing (after rehydration). Wicks were changed four times in the first 4 h of IEF, after which the voltage was maintained at 8000 V (11 cm IPG Ready Strips). Following IEF, IPG Ready Strips were stored at −80°C until used for SDS-PAGE.

Second-dimension SDS-PAGE was performed essentially as described (Burlak *et al*., 2006), except electrophoresis was performed at 50 mA per gel until the dye front reached the bottom of each gel (∼1 h for 11 cm gels). Protein spots were excised and peptides were prepared for ADI-MS/MS analysis as described previously for liquid chromatography-tandem mass spectrometry (LC-MS/MS) (Burlak *et al*., 2006). For simplicity, the combination of IEF and second-dimension SDS-PAGE is abbreviated as 2-DGE.

### Mass spectrometry

Peptide samples (tryptic digests, as described/referenced above) were analysed by automated direct infusion (ADI) using Nanomate (Advion BioSciences, Ithaca, NY), an automated chip-based nano-electrospray interface source, coupled to a quadrupole-time of flight mass spectrometer, QStarXL MS/MS System (Applied Biosystems/Sciex, Framingham, MA). Computer-controlled data-dependent automated switching to MS/MS provided peptide sequence information. AnalystQS software (Applied Biosystems/Sciex) was used for data acquisition. Data processing and databank searching were performed with Mascot software version 2.1 (Matrix Science, Beachwood, OH). The National Center for Biotechnology Information non-redundant protein database (NCBInr, updated 12 May, 2006 at 18:01:48 GMT), National Library of Medicine, NIH was used for the search analysis. Search criteria were limited to double- and triple-charge ions and included monoisotopic masses, analysis of peptides for carbamidomethylation and/or propionamidylation of cysteine, oxidation of methionine, peptide and MS/MS tolerances of 0.2 Da and 0.8 Da respectively, and a maximum of one missed tryptic cleavage. Significance threshold for positive identification was determined by the Mascot Search program.

### Amino acid sequence analysis

Proteins involved in virulence/defence mechanisms, stress response proteins, toxins, haemolysins and proteins of unknown function were evaluated for presence of an LPXTG motif, which predicts a cell wall anchor, and/or for sequences that predict an N-terminal signal peptide or transmembrane region(s). We used the NCBInr database to query the full sequence of each protein identified by ADI/MS/MS for the presence of LPXTG motifs. The presence of membrane spanning domains and N-terminal signal peptide sequences was deduced by searching protein sequences with PSORT software provided by the PSORT WWW server (http://psort.nibb.ac.jp/). Although none of the 20 hypothetical proteins identified in this study contain LPXTG motifs, eight proteins contain probable N-terminal signal peptide sequences and five have predicted transmembrane regions (Table 1 and Table S1).

In addition, sequences of the hypothetical proteins were compared to 902 genomes (Bacterial, Archaea, Viral, and Eukarya) using NCBI and ERGO, a curated NIAID database containing public and proprietary DNA.

### SDS-PAGE and immunoblotting

Proteins were separated by SDS-PAGE, transferred to nitrocellulose membranes, and membranes were blocked with 10% normal goat serum in Tris buffered saline 150 mM NaCL, 50 mM Tris base, pH 7.5, 1% Tween 20 and 0.02% sodium azide (Sigma-Aldrich) overnight at 4°C. Blots were probed with immune or non-immune mouse sera for 1–2 h at ambient temperature or overnight at 4°C. Blots were washed in buffer containing 250 mM NaCl, 10 mM Hepes and 2% Tween 20 (Sigma-Aldrich) and incubated with secondary antibodies conjugated to horseradish peroxidase for 1–2 h at ambient temperature. Immunoreactive proteins were visualized with enhanced chemiluminescence (SuperSignal West Pico, Pierce Biotechnology, Rockford, IL) using Kodak X-Omat film (Eastman Kodak, Rochester, NY).

### Immunofluorescence and confocal laser-scanning microscopy

Acid washed coverslips (No. 1, 13 mm, round) were flamed and coated with 100% normal human serum in 24 well tissue culture plates for 1 h. Coverslips were washed twice with DPBS and synchronized phagocytosis was performed in 24 well plates as described above. Fixed PMNs were washed three times in DPBS and then permeabilized with 0.2% Triton X-100 for 5 min. After three more washes in DPBS, cells were incubated with blocking buffer (DPBS containing 5% BSA, 0.02% sodium azide) for 1 h. Samples were labelled with a 1:1000 dilution of rabbit antiserum containing antibodies specific for Hla (Sigma-Aldrich), SspA, SspB, Aur and 2 μg ml^−1^ of goat polyclonal antibodies specific for human actin-related protein 2 μg ml^−1^ (Santa Cruz Biotechnologies, Santa Cruz, CA) overnight at 4°C. Samples were washed and subsequently labelled with donkey anti-rabbit antibody conjugated with phycoerythrin 1:1000 (Jackson Immunoresearch, West Grove, PA) or donkey anti-goat antibody conjugated to AlexaFluor488 (1:1000) (Molecular Probes, Eugene, OR). PMNs were stained with DAPI (300 nM in DPBS, Molecular Probes) and DRAQ5 (1.25 μM in DPBS, Biostatus Limited, Leicestershire, UK) prior to mounting coverslips onto slides. Slides were analysed with a Zeiss LSM510 confocal laser-scanning microscope coupled to an Axiovert 200M inverted microscope (Carl Zeiss, Thornwood, NY). Images were acquired using a 100× Plan-Apochromat oil immersion objective (1.4 NA) at 512 × 512 pixel resolution with 2.7× digital magnification. Images were adjusted equally for brightness and contrast in Adobe Photoshop CS (Adobe Systems Incorporated, San Jose, CA).

## Supplementary material

The following supplementary material is available for this article online:

Table S1 Inter-strain conservation of proteins of unknown function.

This material is available as part of the online article from http://www.blackwell-synergy.com
