# Supplementary material for: Global analysis of community-associated methicillin-resistant Staphylococcus aureus exoproteins reveals molecules produced in vitro and during infection
Source: Cell Microbiol. 2007 May 1;9(5):1172–90. doi: 10.1111/j.1462-5822.2006.00858.x (PMC2064037; doi:10.1111/j.1462-5822.2006.00858.x)
Supplement: Table S1 — Inter-strain conservation of proteins of unknown function. [file cmi0009-1172_TableS1.doc]

**Table S1.** Inter-strain conservation of proteins of unknown function

| **Unknown proteins** | **Notes** | **LAC (USA300)** | **MW2 (USA400)** | **RF122** | **Mu50** | **N315** | **JH1** | **MSSA476** | **JH9** | **COL** | **MRSA252** |
| --- | --- | --- | --- | --- | --- | --- | --- | --- | --- | --- | --- |
| Hypothetical protein (SAUSA300_0871)***^D1^*** | Possible 2-keto-4-pentenoate hydratase | Query sequence | MW0850 | No Match | SAV0968 | SA0829 | No Match | SAS0838 | No Match | SACOL0973 | SAR0930 |
| Hypothetical protein (SAUSA300_0916)***^D1^*** | Possible 2'-5' RNA ligase | Query sequence | MW0896 | No Match | SAV1015 | SA0873 | No Match | SAS0884 | No Match | SACOL1020 | SAR0985 |
| Hypothetical protein (SAUSA300_1856)***^D2^*** | Putative intracellular protease/amidase | Query sequence | MW1775 | No Match | SAV1835 | SA1653 | No Match | SAS1756 | No Match | SACOL1891 | SAR1926 |
| Hypothetical exported protein (MW0347)***^B6,D7^*** | None | SAUSA300_0372 | Query sequence | SAB0323 | SAV0372 | SA0359 | 90583104 | SAS0349 | 90586673 | SACOL0444 | SAR0390 |
| Hypothetical exported protein (MW2606)***^B1,D1^*** | Similar to YceI of *Escherichia coli* | SAUSA300_2620 | Query sequence | SAB2563 | SAV2687 | SA2479 | 90582018 | SAS2572 | 90584732 | SACOL2711 | SAR2769 |
| Hypothetical protein (MW0395)***^C1,S^*** | Within the Type II νSaα genomic island and exotoxin cluster | SAUSA300_0408 | Query sequence | SAB0387 | SAV0434 | SA0394 | 90583951 | SAS0397 | 90586536 | SACOL0479 | SAR0436 |
| Hypothetical protein (MW0542)***^B1^*** | Possible chlorite dismutase | SAUSA300_0569 | Query sequence | SAB0537 | SAV0587 | SA0544 | 90582689 | SAS0546 | 90585401 | SACOL0633 | SAR0593 |
| Hypothetical protein (MW0819)***^B1,D1,M^*** | None | SAUSA300_0840 | Query sequence | SAB0803 | SAV0937 | SA0798 | 90593980 | SAS0807 | 90585968 | SACOL0940 | SAR0899 |
| Hypothetical protein (MW2068)***^D1,S^*** | Possible NAD-dependent dehydrogenase | ArsR | Query sequence | SAB2028c | SAV2144 | SA1946 | 90583428 | SAS2047 | 90585651 | SACOL2136 | SAR2232 |
| Hypothetical protein (SAUSA300_0408)***^A1,C5,S^*** | Putative surface protein | Query sequence | MW0395 | SAB0387 | SAV0434 | SA0394 | 90583951 | SAS0397 | 90586536 | SACOL0479 | SAR0436 |
| Hypothetical protein SAUSA300_0279****^D1,M,S^*** | Contains a C4-dicarboxylate anaerobic carrier domain | Query sequence | No Match | No Match | No Match | No Match | No Match | No Match | No Match | No Match | No Match |
| Hypothetical protein (MW1884)***^A1,M,S^*** | Within prophage ФSa3 and just upstream of *sak* and *sea* | SAUSA300_1919 | Query sequence | SAB1022 | SAV1942 | SA1754 | 90583206 | SAS1866 | 90586149 | SACOL1169 | SAR2035 |
| Hypothetical protein (MW0577)***^B1,S^*** | None | SAUSA300_0602 | Query sequence | SAB0566 | SAV0613 | SA0570 | 90582663 | SAS0581 | 90585735 | SACOL0669 | SAR0622 |
| Hypothetical protein (MW1786)***^B1,C1,D1^*** | None | SAUSA300_1795 | Query sequence | SAB1778c | SAV1845 | SA1663 | 90583073 | SAS1766 | 90587047 | SACOL1902 | SAR1936 |
| Hypothetical protein (MW1795)***^A2,B1,D2^*** | Possible glucosamine-6-phosphate deaminase | SAUSA300_1804 | Query sequence | SAB1788c | SAV1854 | SA1671 | 90583083 | SAS1777 | 90586790 | SACOL1912 | SAR1945 |
| Hypothetical protein (MW2099)***^B1,D1^*** | Cytosolic protein | SAUSA300_2132 | Query sequence | SAB2054 | SAV2173 | SAS074 | 90583756 | SAS2074 | 90586505 | SACOL2163 | SAR2264 |
| Hypothetical protein (SAUSA300_2327)***^D1^*** | Possible uncharacterized stress protein (general stress protein 26) | Query sequence | MW2302 | SAB2261 | SAV2382 | SA2170 | 90584249 | SAS2272 | 90586852 | SACOL2379 | SAR2469 |
| Hypothetical protein (SAUSA300 pUSA010004)***^C1,M,S^*** | Encoded by plasmid pUSA01 | Query sequence | No Match | No Match | No Match | No Match | No Match | No Match | No Match | No Match | No Match |
| Putative exported protein (MW0355)***^B8,M^*** | Weak homology to ribonucleoside-diphosphate reductase alpha chain in *Streptococcus agalactiae* | SAUSA300_0378 | Query sequence | SAB0329c | SAV0379 | SA0364 | 90583098 | SAS0356 | 90586668 | SACOL0450 | SAR0397 |
| Putative exported protein (MW1757)***^D2,S^*** | Similar to Ear protein. Within the Type II νSaβ genomic island. | SAUSA300_1759 | Query sequence | SAB1675 | SAV1815 | SA1633 | 90583043 | SAS1738 | 90585566 | SACOL1870 | No Match |

*^A^*MW2 supernatants from mid-exponential phase of growth.

*^B^*MW2 supernatants from stationary phase of growth.

*^C^*LAC supernatants from mid-exponential phase of growth.

*^D^*LAC supernatants from stationary phase of growth.

*^E^*Theoretical or predicted.

*^M^*Contains probable transmembrane regions.

*^S^*Contains a probable N-terminal signal peptide sequence.

*^1-8^*Number after the letter *^A,B,C^* or *^D^* indicates number of times a protein was identified.

*The best homology was to Pfl_3008 [*Pseudomonas fluorescens PfO-1*] (ABA74746)
